# Differences between human breast cell lines in susceptibility towards growth inhibition by genistein

**DOI:** 10.1054/bjoc.2001.1980

**Published:** 2001-08

**Authors:** K Dampier, E A Hudson, L M Howells, M M Manson, R A Walker, A Gescher

**Affiliations:** MRC Toxicology Unit, University of Leicester, PO Box 138, Leicester, LE1 9HN, UK; Department of Pathology, University of Leicester, Leicester, UK

**Keywords:** breast cancer, cancer chemoprevention, genistein, soya, malignant cell growth, signal transduction

## Abstract

Genistein is thought to contribute to the putative breast cancer preventive activity of soya. The mechanisms by which it arrests the growth of breast cells are incompletely understood. In order to explore generic features of the modulation of human breast cell growth by genistein, its effects on cell lines MCF-7, ZR-75.1, T47-D, MDA-MB 468, MDA-MB 231 and HBL 100 were compared. Genistein at 1 μM stimulated growth only in MCF-7 cells. At 10 μM it arrested the growth of all 6 cell types, however that of T47-D and HBL 100 cells only in medium with reduced (2%) fetal calf serum. Genistein induced apoptosis in only MDA-MB 468 cells. It arrested cells in the G2 stage of the cell cycle in all cell lines except ZR-75.1. Cells differed in their susceptibility towards inhibition by genistein of phorbol ester-induced proto-oncogene c-fos levels, transcription factor activator protein-1 (AP-1) activity and extracellular signal-regulated kinase (ERK) activity. Genistein augmented anisomycin-induced levels of proto-oncogene c-jun in ZR 75.1 and MCF-7 cells. The results suggest that induction of apoptosis, G2 cell cycle arrest and inhibition of c-fos expression, AP-1 transactivation and ERK phosphorylation may contribute to the growth-inhibitory effect of genistein in some breast cell types, but none of these effects of genistein constitutes a generic mode of growth-arresting action. © 2001 Cancer Research Campaign http://www.bjcancer.com


*British Journal of Cancer* (2001) **85**(4), 618–624
© 2001 Cancer Research Campaign
doi: 10.1054/ bjoc.2001.1980, available online at http://www.idealibrary.com on IDEAL
http://www.bjcancer.com

### Differences between human breast cell lines in susceptibility towards growth inhibition by genistein

**K Dampier[1], EA Hudson[1], LM Howells[1], MM Manson[1], RA Walker[2] and A Gescher[1]**

[1]MRC Toxicology Unit, University of Leicester, PO Box 138, Leicester LE1 9HN, UK; [2]Department of Pathology, University of Leicester, Leicester, UK

**Summary** Genistein is thought to contribute to the putative breast cancer preventive activity of soya. The mechanisms by which it arrests the growth of breast cells are incompletely understood. In order to explore generic features of the modulation of human breast cell growth by genistein, its effects on cell lines MCF-7, ZR-75.1, T47-D, MDA-MB 468, MDA-MB 231 and HBL 100 were compared. Genistein at 1 μM stimulated growth only in MCF-7 cells. At 10 μM it arrested the growth of all 6 cell types, however that of T47-D and HBL 100 cells only in medium with reduced (2%) fetal calf serum. Genistein induced apoptosis in only MDA-MB 468 cells. It arrested cells in the G2 stage of the cell cycle in all cell lines except ZR-75.1. Cells differed in their susceptibility towards inhibition by genistein of phorbol ester-induced proto-oncogene c-fos levels, transcription factor activator protein-1 (AP-1) activity and extracellular signal-regulated kinase (ERK) activity. Genistein augmented anisomycin-induced levels of proto-oncogene c-jun in ZR 75.1 and MCF-7 cells. The results suggest that induction of apoptosis, G2 cell cycle arrest and inhibition of c-fos expression, AP-1 transactivation and ERK phosphorylation may contribute to the growth-inhibitory effect of genistein in some breast cell types, but none of these effects of genistein constitutes a generic mode of growth-arresting action.
© 2001 Cancer Research Campaign http://www.bjcancer.com

**Keywords**: breast cancer, cancer chemoprevention, genistein, soya, malignant cell growth, signal transduction

Breast cancer is the most common malignancy in women in North America and northwestern Europe, with 33 000 new cases diagnosed and 14 000 deaths per year in the UK alone. In contrast, the risk of breast cancer in Asia is only one-quarter of that in the West (Messina et al, 1994; Peterson, 1995). This discrepancy has been associated with differences in dietary habits. For example Asians consume 20–50 times more soy per capita than Westerners (Shao et al, 1998). Soy contains the isoflavones genistein and daidzein in the form of their glycosidic conjugates (Peterson, 1995), and it has been proposed that these isoflavones can prevent breast cancer (Fotsis et al, 1993; Lamartiniere et al, 1995). Genistein at 10–100 μM inhibits the growth and survival of cancer cells derived from the human breast (Zava and Duwe, 1997; Fioravanti et al, 1998; Bail et al, 1998; Hsieh et al, 1998). Growth-inhibitory effects of genistein may be linked to its ability to elicit apoptosis (Shao et al, 1998; Li et al, 1999; Balabhadrapathruni et al, 2000). Paradoxically, genistein at 1–5 μM has been shown to stimulate, rather than attenuate, the growth of MCF-7 breast cancer cells (Zava and Duwe, 1997; Fioravanto et al, 1998; Bail et al, 1998; Hsieh et al, 1998). The mechanisms by which genistein modulates growth and survival of breast cells and prevents breast cancer are not fully understood. As genistein is a phytoestrogen, it may influence cell growth via inhibitory or stimulatory interaction with the oestrogen receptor (Kuiper et al, 1997; 1998). It also possesses antioxidant properties (Barnes and Peterson, 1995) and inhibits DNA topoisomerase II activity (Barnes and Peterson, 1995; Kaufmann, 1998), angiogenesis (Fotsis et al, 1993) and cell cycle regulatory molecules (Shao et al, 1998; Choi et al, 1998), all processes that may be invoked in its chemopreventive activity. Genistein can interfere with cellular signalling by inhibiting the activities of several kinases (Barnes and Peterson, 1995; Kaufmann, 1998), prominent among them epidermal growth factor receptor protein tyrosine kinase (Akiyama et al, 1987). One such pathway involves phosphorylation of mitogen-activated protein kinases (MAPK), such as the ERKs, and culminates in the activation of the transcription factor AP-1 (Karin, 1996; Leppa and Bohnmann, 1999). AP-1 comprises dimers of the c-fos and c-jun protein families (Angel and Karin, 1991; Karin, 1996), and binds to the 12-O-tetradecanoylphorbol-13-acetate (TPA) response element (TRE), a specific target site in the promoters of several genes germane to growth and survival (Smith et al, 1997). Genistein has been shown to attenuate the transcription of c-fos mRNA in breast cancer cells (Schultze-Mosgau et al, 1998) and inhibit both UVB-and TPA-induced c-fos and c-jun expression in mouse skin (Wei et al, 1996; Wang et al, 1998).

Almost all of the published studies on the effect of genistein on breast cell growth and cellular signalling mechanisms have focussed on a small number of cell types, in particular on MCF-7 cells. The study described here was designed to explore generic features pertinent to the modulation of breast cell growth by genistein using a panel of 6 cell types, and to highlight differences between cell lines in their response to genistein. To that end effects of genistein on the following human breast cell types were compared: MCF-7, ZR-75.1, T47-D, MDA-MB 468 and MDA-MB 231 cells, all of which are malignant, and HBL 100 cells, which are immortalized but not malignant. Furthermore we wished to relate differences between cells in their growth response to genistein with their susceptibility towards genistein-induced biochemical changes that may contribute to cell growth inhibition, such as changes in cell cycle regulation, AP-1 activity and levels of c-fos and c-jun proteins.

*Received 6 December 2000*
*Revised 22 May 2001*
*Accepted 24 May 2001*

*Correspondence to:* A Gescher

### MATERIAL AND METHODS

### Cell lines and chemicals

HBL 100, MCF-7, ZR-75.1, T47-D, MDA-MB 468 and MDA-MB 231 cells were originally obtained from the American Type Culture Collection (Manassas, VA, USA). Media were purchased from Gibco Life Technologies (Paisley, UK). Genistein, 12-O-tetradecanoylphorbol-13-acetate (TPA) and anisomycin were purchased from Sigma-Aldrich Company Ltd. (Poole, UK). Genistein and TPA were dissolved in dimethylsulphoxide (DMSO), aliquoted and frozen until use. Control incubates contained DMSO only, and at the concentrations used (at or below 0.01%) DMSO did not interfere with growth. FuGene transfection reagent was purchased from Boehringer Mannheim (Lewes, UK). The antibodies for phosphorylated ERK (pERK), c-fos and c-jun were obtained from Santa Cruz Biotechnology (Santa Cruz, CA, USA). The annexin V reagent was obtained from Bender MedSystems (Vienna, Austria). The antibody for poly (ADP-ribose)-polymerase (PARP) and N-acetyl-Asp-Glu-Val-Asp 7-amido-4-trifluoromethyl coumarin (DEVD.afc) enzyme substrate (used for the determination of apoptosis) was kindly provided by Dr M. MacFarlane (MRC Toxicology Unit, University of Leicester), and glutathione-S-transferase (GST)-c-jun protein (for measurement of c-jun N-terminal kinase [JNK] activity) was a gift from Dr M. Dickens (Department of Biochemistry, University of Leicester, UK).

### Cell growth studies

Cells were routinely passaged in the following media, all supplemented with 10% fetal calf serum (FCS): RPMI-1640 and 2 mM glutamax (MDA-MB 468 and MCF-7), DMEM/F12 (Ham) 1:1 containing 2 mM glutamax, 15 mM HEPES (ZR-75.1), DMEM containing 1 g $l^{-1}$ glucose, 2 mM glutamax, 110 mg $l^{-1}$ sodium pyruvate (HBL 100, T47-D and MDA-MB 231). All cell lines tested negative for mycoplasma infection. For studies of the effect of genistein on growth, cells seeded at $2 \times 10^4$ per well (17.5 mm) were grown in media supplemented with FCS (10% or 2%). After a 48-hour attachment period, cells were incubated with genistein (1 μM or 10 μM) for 96 h and subsequently counted with a Coulter Counter ZM (Beckman Coulter, Luton, UK).

### Flow-cytometric analysis

For cell cycle investigations, cells (1 or $2 \times 10^6$ per 35 mm well) were incubated with genistein (10 μM) for 96 h after a 24-hour attachment period in medium with 2% FCS. Cells were harvested by trypsinization, washed with PBS, then pellets were resuspended in ethanol (70%, 2 ml) and incubated for 3 h at 4˚C. RNase (0.1 mg) and propidium iodide (5 μg) were added, and cells were kept for 24 h (4˚C) (Rickwood and Hames, 1990). Flow cytometric analysis was performed using a FACScan flow cytometer (Becton Dickinson, San Jose, CA, USA) with Cell Quest software.

### Measurement of apoptosis

For assessment of apoptosis, cells (1 or $2 \times 10^6$ per 35 mm well) were incubated with genistein (10 μM) for 96 h after a 24-hour attachment period in medium with 10% FCS. Cells obtained by trypsinization were combined with those that had spontaneously detached during the incubation. Apoptosis was assessed in 3 ways. To determine phosphatidylserine externalization by annexin V staining, cells were pelleted and suspended in annexin buffer (1 ml; 10 mM HEPES pH 7.4, 150 mM NaCl, 5 mM KCl, 1 mM $MgCl_2$, 1.8 mM $CaCl_2$). Annexin V was added to cells (final concentration 100 ng $ml^{-1}$), and cells were incubated for 8 min at room temperature, after which propidium iodide (1.5 μg) was added, and cells were analysed by flow cytometry.

To measure PARP cleavage (Germain et al, 1999), the cell pellet was washed with PBS, snap-frozen in liquid nitrogen and thawed at 37˚C 3 times in succession. The resulting pellet was resuspended in sample buffer. Samples were sonicated and subjected to SDS-PAGE as described (Sambrook et al, 1989). Membranes were blocked in 5% non-fat milk in Tris-buffered saline with Tween-20 (TBS-T) for 1 h at room temperature and incubated for 1 h with a PARP-specific antibody (1:10 000 dilution). For detection the enhanced chemiluminescence (ECL) detection system (Amersham Life Science Ltd, Little Chalfont, UK) was used.

To measure caspase-3 activity (MacFarlane et al, 1997), cells were harvested as for analysis of PARP cleavage, but resuspended in PIPES buffer. Extracts containing 50 μg protein were combined with a 1.25 ml assay buffer and DEVD.afc enzyme substrate (final concentration 2.5 mM). The samples were assayed for caspase-3 activity using a luminescent spectrophotometer (Perkin Elmer LS 50B, Beaconsfield, UK). Results were calculated as units of enzyme activity (pmol $mg^{-1}$ protein $min^{-1}$) using UV WinLab software.

### Measurement of AP-1 transactivation

The TPA response element reporter construct (TRE-pGL2), kindly provided by Professor P. Parker (ICRF, London, UK), contained 3 tandem TRE sites upstream of a luciferase coding region, and was transfected into cells using electroporation or FuGene. In the case of electroporation, $8 \times 10^7$ cells were transfected with the TRE-pGL2 reporter construct (2.5 pmol), together with a control expression vector containing the β-galactosidase gene driven by the human cytomegalovirus immediate early gene promoter, pCMVβ (0.075 pmol). Cells were allowed to recover in medium containing 10% FCS for 5 h, after which they were serum starved for 24 h. Transfections with FuGene involved serum starvation of cells ($3 \times 10^6$ per 35 mm well) for 24 h, followed by incubation for 24 h with FuGene, TRE-pGL2 construct (0.29 pmol) and pCMVβ (0.23 pmol). After transfection by either method, cells were exposed to genistein (1, 10 or 50 μM) for 1 h, prior to incubation with TPA (0.2 μM) for 4 h. Luciferase and β-galactosidase enzyme activites were determined using Promega assay kits and a Wallac Microbeta 1450 plate reader, or Labsystems iEMS plate reader, respectively. The expression of β-galactosidase was used to control for transfection efficiency by normalizing all results to expression of this gene.

### Western blot analysis of phosphorylated ERKs and fos/jun proteins and measurement of JNK activity

Cells were seeded at 2–4 $\times 10^6$ per well (35 mm), allowed to adhere, serum starved for 24 h and treated with genistein (50 μM) for 30 min prior to inclusion in the medium for 2 h of either TPA (0.2 μM) in the case of analysis of c-fos levels and ERK phosphorylation, or anisomycin (100 nM) for analysis of c-jun and JNK activity. Proteins were extracted either from whole cells for the

© 2001 Cancer Research Campaign 

determination of ERK phosphorylation and JNK activity or from isolated nuclei for analysis of c-fos and c-jun levels. For Western analysis, proteins were electrophoretically separated on a 10% polyacrylamide gel and transferred to nitrocellulose (Hybond, Amersham Life Science Ltd) using a wet blotting system. For analysis of ERKs, membranes were blocked in 5% bovine serum albumin, and for analysis of c-fos and c-jun, in 10% non-fat milk and TBS-T at 4˚C for 24 h. Membranes were then incubated with antibodies specific for either pERK (1:2000 dilution) for 90 min or c-fos or c-jun (1:2000 dilution) for 2 h. Membranes were washed with TBS-T, incubated with a secondary antibody conjugated with horseradish peroxidase, and the signal was detected using the ECL detection system. Scanning and densitometry were performed using a Molecular Dynamics Densitometer and quantified by Image Quant Analysis software.

After treatment with anisomycin and/or genistein, protein extracts of ZR-75.1 and MCF-7 cells were subjected to a JNK kinase assay as described by Croisy–Delcey et al (1997). Phosphorylation of GST-c-jun was analysed by SDS PAGE. Gels were transferred onto blotting paper, dried at 65˚C for 1 h and visualized by autoradiography after incubation for 6 h at –80˚C. Scanning and densitometry were performed using a Molecular Dynamics PhosphorImager and quantified by Image Quant Analysis software.

### Statistical analysis

Statistical significance was assessed using the ANOVA General Linear Model (Afifi and Azen, 1979) followed by Fisher's least significant difference posthoc test (Snedecor and Cochran, 1980) or one-way ANOVA followed by Tukey's posthoc test.

### RESULTS

### Effect of genistein on cell growth and survival and cell cycle distribution

Human-derived breast cells were incubated with genistein at 1 μM or 10 μM. At the low concentration, genistein accelerated the growth of MCF-7 cells, increasing cell numbers by 40% over controls. This mitogenic effect of genistein was not seen in any of the other 5 cell types (Figure 1). At the higher concentration, genistein inhibited the growth of MCF-7, MDA-MB 468, ZR 75.1 and MDA-MB 231 cells, but not of HBL 100 and T47-D cells, when cells were cultured with medium containing 10% FCS (Figure 1A). In order to determine whether constituents of the medium influence cell growth modulation by genistein, its effect on cell numbers was reinvestigated under conditions of low serum concentration (2%). The effects of genistein on the growth of cells

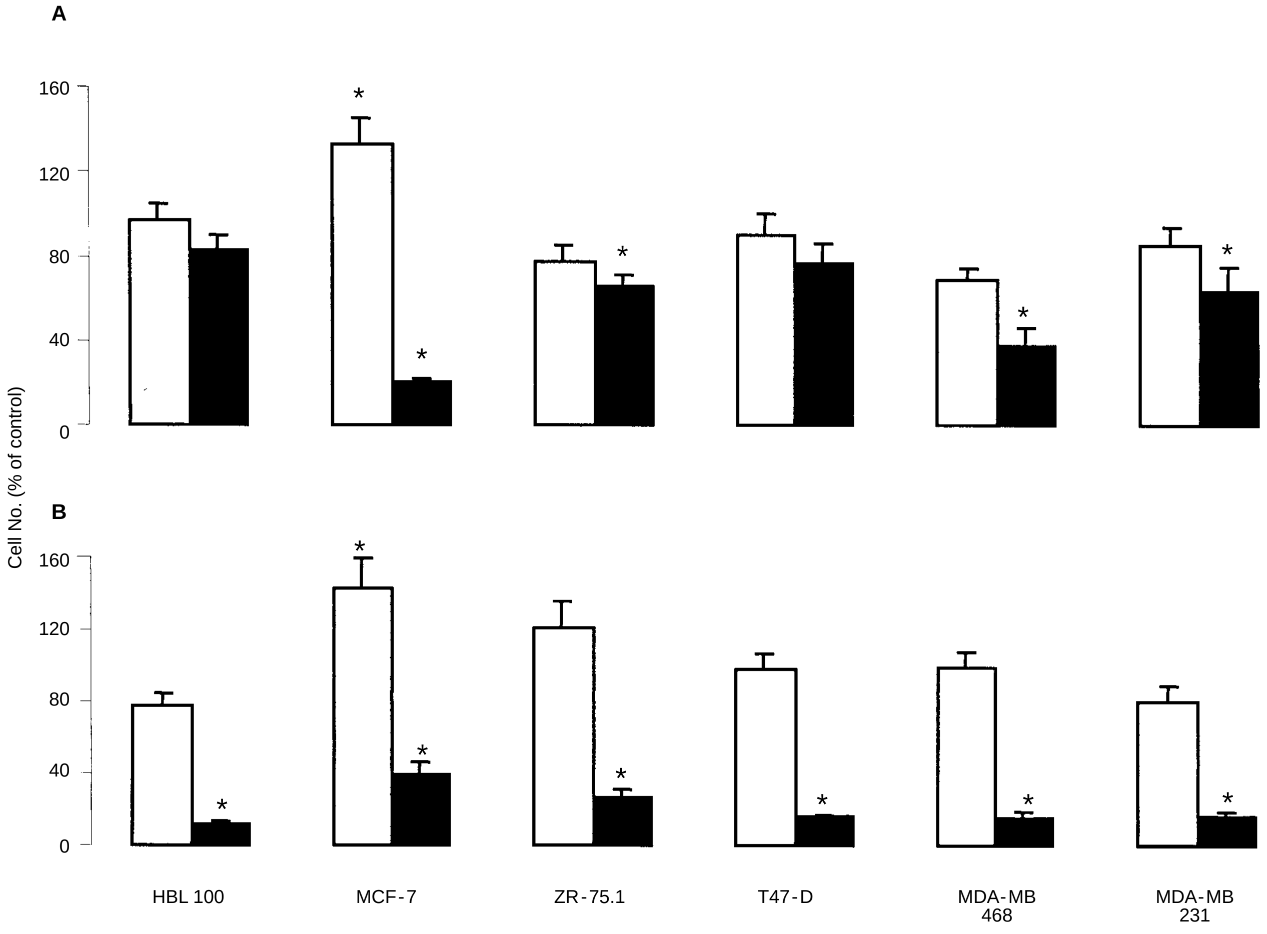

**Figure 1** Effect of genistein 1 μM (open bars) and 10 μM (closed bars) on the growth of breast cells cultured for 96 h in medium with 10% FCS (**A**) or 2% FCS (**B**). Results, which are presented as percentage of cells in control cultures, are the mean ± SEM of 3 independent determinations, each conducted in duplicate. Asterisks indicate that values are significantly different from controls ($P < 0.05$, two-way ANOVA followed by Fisher's least significant difference posthoc test). For details of culture conditions, see Materials and methods

 © 2001 Cancer Research Campaign cultured under these conditions differed from that observed under normal culture conditions (10% FCS), in that genistein (10 μM) inhibited cell numbers significantly also in HBL 100 and T47-D cells and was a more potent inhibitor of the growth of MDA-MB 468, ZR-75.1 and MDA-MB-231 cells (Figure 1B).

The hypothesis that genistein-induced growth arrest was related to induction of apoptosis was tested. Consistent with previous reports (Li et al, 1999; Balabhadrapathruni et al, 2000), genistein induced apoptosis in MDA-MB 468 cells grown in media with either 10% or 2% FCS, as borne out by measurement of annexin staining, PARP cleavage and caspase-3 activity after incubation for 96 h. The percentage of apoptotic cells as adjudged by annexin staining in cells incubated in medium with 10% FCS and genistein (10 μM) rose from 5% in controls to 19.5%. Analysis of PARP protein by Western blot showed a decrease in PARP protein band intensity of 38% in cells exposed to genistein, as compared to control cells, suggesting genistein-induced PARP degradation in these cells. Furthermore, genistein increased caspase-3 enzyme activity 5-fold over that in control cells (results not shown). In the other 5 breast cell types, genistein did not cause changes indicative of apoptosis, although it increased the necrotic cell population slightly.

The mitogenic effect of genistein (1 μM) in MCF-7 cells was not accompanied by any significant change in cell cycle distribution. However, genistein (10 μM) blocked cell cycle progression in the G2/M phase in 5 of the 6 cell types grown in 2% FCS (Table 1). ZR 75.1 cells were insensitive towards the G2/M phase blockade elicited by genistein, but unlike the other cells they accumulated somewhat in G1.

### Effect of genistein on TPA-induced AP-1 transactivation, ERK phosphorylation and levels of c-fos and c-jun

Cells were transfected with a TRE-pGL2 reporter construct, the activity of which reflects AP-1 transactivation, as it contains 3 TRE sites upstream of a luciferase gene. TPA increased luciferase activity 1.5- to 10-fold over control values in HBL 100, ZR-75.1, MDA-MB 468 and T47-D, but failed to induce AP-1 activity in MCF-7 and MDA-MB 231 cells. TPA was without effect in cells into which the empty vector had been transfected. The effect of genistein (1–50 μM) on TPA-induced luciferase expression was examined. Genistein reduced luciferase activity only in MDA-MB 468 and T47-D cells (results not shown), albeit the extent of reduction was variable and not significant, and it was observed only at the highest genistein concentration. In all of these experiments Fugene was used to transfect the reporter construct into cells. The inhibitory activity of genistein was confirmed in experiments in which the TRE-pGL2 reporter construct was transfected into MDA-MB 468 cells using electroporation. In these cells the extent of TPA-induced luciferase expression was similar to that observed in cells transfected by Fugene, and genistein (50 μM) decreased it significantly by 75 ± 2% (mean ± SD; $n$ = 3). Paradoxically, in ZR 75.1 cells genistein (50 μM) augmented, rather than decreased, TPA-induced luciferase expression, even though this elevation was highly variable. In the absence of TPA, genistein did not augment AP-1 activity.

Changes in AP-1 activity can be mediated by alterations of elements of the MAPK cascade via the ERKs. The effect of genistein on TPA-induced ERK 1 and 2 phosphorylation was studied using Western blot analysis. TPA induced ERK 1 and 2 phosphorylation in T47-D, ZR-75.1 (Figure 2A) and MCF-7 cells (result not shown). Such induction was not reproducibly observed in HBL 100, MDA-MB 468 or MDA-MB 231 cells. Genistein (50 μM) significantly inhibited TPA-induced ERK phosphorylation in ZR-75.1 and T47-D cells, by 70% and 47%, respectively (Figure 2A), but not in MCF-7 cells (results not shown).

To examine the mechanism by which genistein regulates AP-1 transactivation, its effects on levels of c-fos and c-jun, major constituents of the AP-1 complex, were studied. Expression of c-fos and c-jun was stimulated by incubation of cells with TPA and anisomycin, respectively. Genistein (50 μM) decreased c-fos levels in MDA-MB 468 and T47-D cells by 77% and 73%, respectively (Figure 2B). In contrast, genistein stimulated levels of c-jun over those elicited by anisomycin 2.5 and 3-fold in MCF-7 and ZR-75.1 cells, respectively (Figure 2C). Genistein on its own in the absence of anisomycin did not affect c-jun levels (results not shown). It was also devoid of any effect on induced c-fos or c-jun levels in HBL 100 and MDA-MB 231 cells, on c-fos in MCF-7 and ZR-75.1 cells or on c-jun in MDA-MB 468 and T47-D cells

**Table 1** Effect of genistein (10 μM) on cell cycle distribution of breast cells cultured in 2% FCS

| Cell type | Treatment | Cell cycle distribution* | | |
|---|---|---|---|---|
| | | G1 | S | G2/M |
| HBL 100 | Control | 61.8 ± 3.1 | 13.8 ± 3.6 | 20.2 ± 3.1 |
| | Genistein | 34.4 ± 16.5† | 13.7 ± 2.1 | 45.5 ± 13.8* |
| MCF-7 | Control | 78.1 ± 7.7 | 9.6 ± 2.9 | 8.5 ± 3.1 |
| | Genistein | 79.2 ± 2.4 | 9.2 ± 2.4 | 22.0 ± 4.2* |
| MDA-MB 468 | Control | 72.0 ± 6.0 | 11.1 ± 2.8 | 13.7 ± 6.1 |
| | Genistein | 62.2 ± 13.0 | 11.2 ± 2.8 | 43.3 ± 18.6* |
| ZR-75.1 | Control | 77.9 ± 8.8 | 8.8 ± 4.9 | 9.9 ± 2.6 |
| | Genistein | 86.2 ± 5.3 | 4.8 ± 2.4 | 7.7 ± 2.3 |
| T47-D | Control | 76.7 ± 2.5 | 8.8 ± 1.4 | 11.5 ± 4.2 |
| | Genistein | 65.8 ± 10.5* | 7.3 ± 1.4 | 20.9 ± 8.3* |
| MDA-MB 231 | Control | 74.6 ± 2.9 | 6.3 ± 2.3 | 17.3 ± 1.7 |
| | Genistein | 63.3 ± 25.6* | 9.2 ± 4.0 | 25.9 ± 6.0* |

*Results are presented as percentage values and are the mean ± SEM of four separate experiments, each conducted in duplicate. †Significantly different from control cells ($P$ < 0.05, general linear model ANOVA, followed by Fisher's least significant difference posthoc test).

© 2001 Cancer Research Campaign 

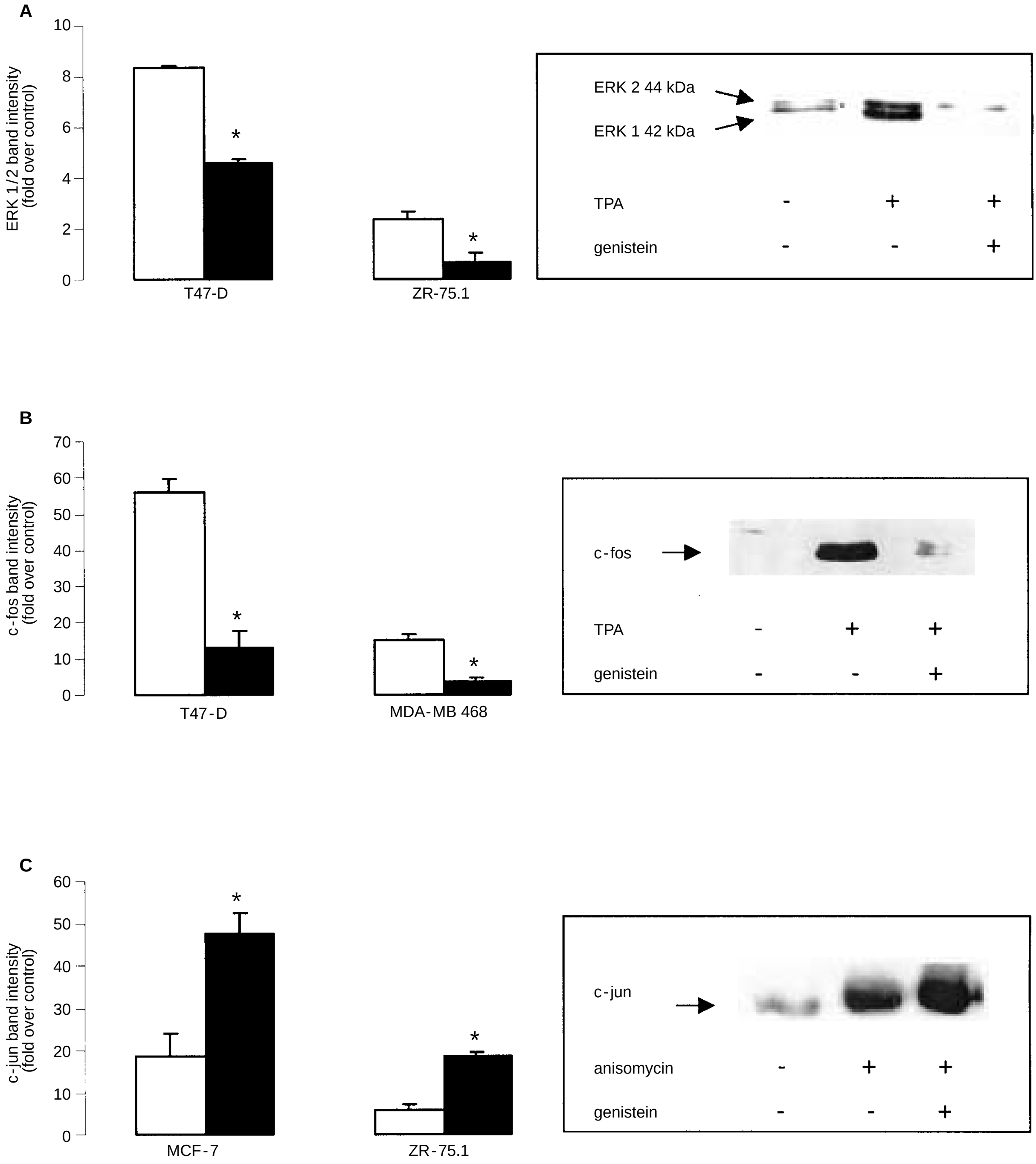

**Figure 2** Effect of genistein (50 μM) on (**A**) TPA-induced ERK phosphorylation in T47D and ZR-75.1 cells, with a representative blot for ZR-75.1 cells, (**B**) TPA-induced c-fos levels in T47-D and MDA-MB 468, with a representative blot for MDA-MB 468 cells, and (**C**) anisomycin-induced c-jun levels in MCF-7 and ZR-75.1 cells, with a representative blot for ZR-75.1 cells. Values were derived by laser densitometry from Western blot analyses. Genistein alone did not affect c-jun levels. Results are presented as fold changes in band density compared to control incubates. Open bars represent values after treatment with TPA (0.2 μM) (**A, B**) or anisomycin (100 μM) (**C**) only. Closed bars represent values after treatment with TPA (**A, B**) or anisomycin (**C**), plus genistein. Results are the mean ± SEM of 3 independent experiments. Asterisks indicate that values are significantly different from those obtained in the presence of TPA or anisomycin only ($P < 0.05$, two-way ANOVA followed by Fisher's least significant difference posthoc test). For details of incubation and Western analysis see Materials and methods

(results not shown). In order to assess the effect of genistein on JNK activity in the 2 cell types in which genistein increased c-jun protein levels, its effect on anisomycin-induced c-jun phosphorylation was studied. Anisomycin induced JNK phosphorylating activity by a factor of 5.5 ± 1.2 over controls in ZR-75.1 cells, and by 3.5 ± 0.7 in MCF-7 cells ($n = 3$). Genistein (50 μM) reduced it back to control levels in ZR-75.1 cells but not in MCF-7 cells (results not shown).

 © 2001 Cancer Research Campaign

**Table 2** Effects of genistein on breast-derived cell types

| | Cell type | | | | | |
|---|---|---|---|---|---|---|
| | **HBL 100** | **MCF-7** | **MDA 468** | **ZR-75** | **T47-D** | **MDA 231** |
| Growth inhibition* | –[†‡] | + | + | + | –[‡] | + |
| Apoptosis* | – | – | + | – | – | – |
| G2/M arrest[§] | + | + | + | – | + | + |
| G1 arrest[§] | – | – | – | + | – | – |
| AP-1 transactivation (+TPA only)[¶] | ↑ | – | ↑ | ↑ | ↑ | – |
| AP-1 transactivation (+ TPA and genistein)** | – | – | ↓ | ↑ | ↓ | – |
| c-fos levels (+TPA and genistein)** | – | – | ↓ | – | ↓ | – |
| c-jun levels (+ anisomycin and genistein)** | – | ↑ | – | ↑ | – | – |
| ERK 1/2 phosphorylation (+TPA only)[¶] | – | ↑ | – | ↑ | ↑ | – |
| ERK 1/2 phosphorylation (+ TPA and genistein)** | – | – | – | ↓ | ↓ | – |

*Effect of genistein (10 μM) in cells maintained in 10% FCS; [†]–: no effect, +: effect, ↑: upregulation, ↓: downregulation; [‡]Note that the growth of HBL 100 and T47-D cells was inhibited by genistein when cells were cultured with 2% rather than 10% FCS; [§]Effect of genistein(10 μM) in cells maintained in 2% FCS; [¶]Effect of TPA alone; **Effect of genistein (50 μM) after stimulation by TPA or anisomycin. For details see results and discussion.

### DISCUSSION

The results presented earlier show that the growth of all 6 human breast cell lines investigated in this study was affected by genistein at 10 μM, when cells were cultured in medium containing only 2% serum. Nevertheless there were differences between cells in terms of their sensitivity towards genistein. The enhancement of breast cell growth by genistein (1 μM), as seen in MCF-7 cells, is not a generic feature of human breast cells. The observation that genistein in the $10^{-6}$ M range promotes the growth of MCF-7 has raised concern that physiologically achievable concentrations of genistein might support, rather than counteract, the progression of breast cancer in women with pre-diagnostic neoplastic changes (Zava and Duwe, 1997; Fioravanti et al, 1998; Bail et al, 1998; Hsieh et al, 1998). Such an effect would severely confound any potential benefit of genistein consumption. Growth promotion has been associated with the interaction of genistein with ER-α and -β (Kuiper et al, 1997; 1998). A review of the relevant, partially contradictory and confusing literature (Enmark et al, 1997; Nakatani et al, 1999; Shao et al, 1998; Fioravanti et al, 1998; Dotzlaw et al, 1996) and complementary unpublished observations in this laboratory (Dampier, 2001) regarding levels of ER-α protein and ER-β mRNA suggest tentatively that HBL-100, MDA-MB 231 and MDA-MB 468 cells have ER-β, ZR-75.1 and T47-D cells possess ER-α, and MCF-7 cells have both receptors. This comparison renders the possibility unlikely that the growth promotion caused by genistein specifically in MCF-7 cells is intrinsically linked to expression of either ER-α or -β.

The notion that divergent mechanisms mediate the growth-arresting efficacy of genistein in the different breast cell lines is supported by the following 4 pieces of evidence:

1. The susceptibility of the cells towards growth inhibition by genistein was differentially affected by the serum content of the medium.
2. Genistein caused apoptosis in MDA-MB 468 cells, but not in MCF-7, ZR-75 or MDA 231 cells, which were similarly susceptible to genistein-induced growth arrest as MDA-MB 468.
3. Under culture conditions with low FCS (2%) genistein significantly arrested cells in the G2 phase of the cell cycle in 5 of the 6 cell lines, but not ZR-75.1, in which growth arrest was accompanied by a slight increase in the G1 cell population.
4. Cells differed considerably in their susceptibilities towards inhibition by genistein of elements of TPA-induced cell signalling: AP-1 activity was inhibited only in MDA-MB 468 and T47-D cells, ERK activation only in ZR 75.1 and T47-D cells, and c-fos levels only in MDA-MB 468 and T47-D cells.

Some of these observations can be rationalized in terms of mechanistic connectivity or complementarity. Table 2 summarizes some of the effects of genistein on growth, apoptosis, cell cycle distribution, AP-1 activity, ERK phosphorylation, and c-fos and c-jun protein levels in the cell lines studied. In ZR-75.1 cells genistein appeared to increase, rather than decrease, tumour promoter-mediated AP-1 activity. Compatible with this observation, genistein increased c-jun protein levels in the presence of the jun activator anisomycin. However this increase was accompanied by a decrease in anisomycin-induced JNK activity. So, the functional consequences of the contrasting changes elicited by genistein with respect to c-jun protein on the one hand and JNK activity on the other are difficult to predict. In T47-D cells, the decrease of ERK phosphorylation is compatible with the attenuation of both c-fos protein levels and AP-1 activity. This coincidence suggests that in these cells genistein may inhibit growth by interfering with MAPK signalling, which in turn abrogates AP-1 transcription via attenuation of c-fos expression. In MDA-MB 468 cells, the observed reduction by genistein of c-fos protein expression, compatible with the effect of genistein on c-fos transcription reported previously (Schultze-Mosgau et al, 1998), explains the amelioration of AP-1 activity. These events might contribute to the growth-inhibitory and apoptosis-inducing effects of genistein in MDA-MB 468 cells. Undoubtedly the concentrations of genistein that have been reported here and by others to elicit growth arrest and cause changes in signalling, are higher than the 3–4 μM reported to be achieved in the plasma of individuals who consume large amounts of soya (Adlercreutz et al, 1995; Barnes and Peterson, 1995). Nevertheless, as genistein and related isoflavanoid molecules have recently been discussed as potential chemopreventive or chemotherapeutic agents in their own right and not only as

© 2001 Cancer Research Campaign constituents of the food matrix, it is conceivable that they could be administered clinically at much higher doses than those associated with dietary polyphenol consumption, thus potentially achieving target tissue concentrations in the $10^{-5}$–$10^{-4}$ M range.

In conclusion, our findings suggest that whilst genistein at higher concentrations consistently arrests the growth of breast cells when they are cultured under low serum conditions, susceptibility to growth promotion induced by genistein at low concentrations is not a generic feature of cells derived from this tissue. The results are consistent with the notion that induction of apoptosis, G2 cell cycle arrest and inhibition of components of MAPK signalling, c-fos protein expression and AP-1 transactivation may contribute to the growth-inhibitory effect of genistein in some breast cell types. None of these effects of genistein constitutes a predominant mode of growth-arresting action as they do not occur consistently in all breast cell lines. Therefore this study does not suggest a unifying picture of the molecular events exerted by genistein. Instead, the relative importance of the individual components of the heterogeneous spectrum of mechanisms associated with growth modulation by this isoflavone is probably breast cell type specific.

### ACKNOWLEDGEMENTS

We would like to thank Dr M. MacFarlane (MRC Toxicology Unit) for provision of the PARP antibody and DEVD substrate, Professor P. Parker (ICRF Laboratories, London, UK) for the TRE construct, Dr M. Dickens (Department of Biochemistry, University of Leicester, UK) for the GST-c-jun protein and Drs M. MacFarlane and S. Plummer (MRC Toxicology Unit) for help with some of the experiments.

### REFERENCES

Adlercreutz HCT, Goldin B, Gorbach SL, Hockerstedtk AV, Watanabe S, Hamalainen EK, Markkanen MH, Makela TH, Wahala KT, Hase TA and Fotsis T (1995) Soybean phytoestrogen intake and cancer risk. *J Nutr* **125**: 757S–767S

Afifi AA and Azen SP (1979) The analysis of variance. In: Afifi AA and Azen SP (eds), *Statistical Analysis: A Computer Orientated Approach*, 2nd edn, pp. 198–273. Academic Press Inc.: London

Akiyama T, Ishida J, Nakagawa S, Ogawara H, Watanabe S, Itck N, Shibuiya M and Fukami Y (1987) Genistein, a specific inhibitor of tyrosine-specific protein kinases. *J Biol Chem* **262**: 5592–5595

Angel P and Karin M (1991) The role of Jun, Fos and the AP-1 complex in cell proliferation and transformation. *Biochim Biophys Acta* **1072**: 129–157

Bail JC, Varnat F, Nicolas JC and Habrioux G (1998) Estrogenic and antiproliferative activities on MCF-7 human breast cancer cells by flavanoids. *Cancer Lett* **130**: 209–216

Balabhadrapathruni S, Thomas TJ, Yurkow EJ, Amenta PS and Thomas T (2000) Effects of genistein and structurally related phytoestrogens on cell cycle kinetics and apoptosis in MDA-MB-468 human breast cancer cells. *Oncol Reports* **7**: 3–12

Barnes S and Peterson TG (1995) Biochemical targets of the isoflavone genistein in tumor cell lines. *Proc Soc Exp Biol Med* **208**: 103–107

Choi YH, Zhang L, Lee WH and Park K-Y (1998) Genistein-induced G2/M arrest is associated with the inhibition of cyclin B1 and the induction of p21 in human breast carcinoma cells. *Int J Oncol* **13**: 391–396

Croisy-Delcey M, Croisy A, Mousset S, Letourneur M, Bisagni E, Jacquemin-Sablon A and Pierre J (1997) Genistein analogues: effects on epidermal growth factor receptor tyrosine kinase and on stress-activated pathways. *Biomed Pharmacother* **51**: 286–294

Dampier K (2001) Studies of the mechanisms of action of the chemopreventive agent genistein. PhD thesis, University of Leicester.

Dotzlaw H, Leygue E, Watson P and Murphy LC (1996) Expression of estrogen receptor-β in human breast tumors. *J Clin Endocrinol Metab* **82**: 2371–2374

Enmark E, Pelto-Huikko M, Grandien K, Lagercrantz S, Lagercrantz J, Fried G, Nordenskjold M and Gustafsson J (1997) Human estrogen receptor-β gene structure, chromosomal localization and expression pattern. *J Clin Endocrinol Metab* **82**: 4258–4265

Fioravanti L, Cappelletti V, Miodini P, Ronchi E, Brivio M and Di Fronzo G (1998) Genistein in the control of breast cancer cell growth: insights into the mechanism of action *in vitro*. *Cancer Lett* **130**: 143–152

Fotsis T, Pepper M, Adlercreutz H, Fleischmann G, Hase T, Montesano R and Schweigerer L (1993) Genistein, a dietary-derived inhibitor of *in vitro* angiogenesis. *Proc Natl Acad Sci USA* **90**: 2690–2694

Germain M, Affar E-B, D'Amours D, Dixit VM, Salveseni GS and Poirier GG (1999) Cleavage of automodified poly (ADP-ribose) polymerase during apoptosis. *J Biol Chem* **274**: 28379–28384

Hsieh C, Santell RC, Haslam SZ and Helferich WG (1998) Estrogenic effects of genistein on the growth of estrogen receptor-positive human breast cancer (MCF-7) cells *in vitro* and *in vivo*. *Cancer Res* **58**: 3833–3838

Karin M (1996) The regulation of AP-1 activity by mitogen-activated protein kinases. *Philos Trans R Soc Lond B* **351**: 127–134

Kaufmann WK (1998) Human topoisomerase II function, tyrosine phosphorylation and cell cycle checkpoints. *Proc Soc Exp Biol Med* **217**: 327–334

Kuiper GGJM, Carlsson B, Grandien K, Enmark E, Haggblad J, Nilsson S and Gustafsson J-A (1997) Comparison of the ligand binding specificity and transcript tissue distribution of estrogen receptors α and β. *Endocrinology* **138**: 863–870

Kuiper GGJM, Lemmen JG, Carlsson B, Corton JC, Safe SH, Saag PT, Burg B and Gustafsson J-A (1998) Interaction of estrogenic chemicals and phytoestrogens with estrogen receptor β. *Endocrinology* **139**: 4252–4263

Lamartiniere CA, Moore J, Holland M and Barnes S (1995) Neonatal genistein chemoprevents mammary cancer. *Proc Soc Exp Biol Med* **208**: 120–123

Leppa S and Bohnmann D (1999) Diverse functions of JNK signalling and c-jun in stress response and apoptosis. *Oncogene* **18**: 6158–6162

Li Y, Upadhyay S, Bhuiyan M and Sarkar FH (1999) Induction of apoptosis in breast cancer cells MDA-MB-231 by genistein. *Oncogene* **18**: 3166–3172

MacFarlane M, Cain K, Sun X, Alnemri ES and Cohen GM (1997) Processing/activation of at least four interleukin 1 β-converting enzyme-like proteases during the execution phase of apoptosis in human monocytic tumor cells. *J Cell Biol* **137**: 469–479

Messina MJ, Persky V, Setchell KDR and Barnes S (1994) Soy intake and cancer risk: a review of the *in vitro* and *in vivo* data. *Nutr Cancer* **21**: 113–127

Nakatani K, Thompson DA, Barthel A, Sakaue H, Liu W, Weigel RJ and Roth RA (1999) Upregulation of Akt3 in estrogen receptor-deficient breast cancers and androgen-independent prostate cancer lines. *J Biol Chem* **274**: 21528–21532

Peterson G (1995) Evaluation of the biochemical targets of genistein in tumor cells. *J Nutr* **125**: 784S–788S

Rickwood D and Hames BD (1990) *Flow Cytometry. A Practical Approach*, p. 75. Oxford University Press: Oxford

Sambrook J, Fritsch EF and Maniatis T (1989) SDS-Polyacrylamide gel electrophoresis of proteins. In: Sambrook J, Fritsch EF and Maniatis T (eds) *Molecular Cloning: A Laboratory Manual* pp 18– 47. Harbor Laboratory Press: Cold Spring

Schultze-Mosgau M-H, Dale IL, Gant TW, Chipman JK, Kerr DJ and Gescher A (1998) Regulation of c-fos transcription by chemopreventative isoflavanoids and lignans in MDA-MB 468 breast cancer cells. *Eur J Cancer* **34**: 1425–1431

Shao Z-M, Alpaugh ML, Fontana JA and Barsky SH (1998) Genistein inhibits proliferation similarly in estrogen receptor positive and negative human breast carcinoma cell lines, characterised by $p21^{WAF1/CIP1}$ induction, G2/M arrest and apoptosis. *J Cell Biochem* **69**: 44–54

Smith LM, Birrer MJ, Stampfer MR and Brown PH (1997) Breast cancer cells have lower activating protein-1 transcription factor activity than normal mammary epithelial cells. *Cancer Res* **57**: 3046–3054

Snedecor GW and Cochran WG (1980) Analysis of variance. In: Snedecor GW and Cochran WG (eds) Statistical Methods, 7th edn, pp 215–237. Iowa State University Press: Ames

Wang Y, Zhang X, Lebwohl M, Deleo V and Wei H (1998) Inhibition of ultraviolet B (UVB)-induced c-fos and c-jun expression *in vivo* by a tyrosine kinase inhibitor genistein. *Carcinogenesis* **19**: 649–654

Wei H, Barnes S and Wang Y (1996) Inhibitory effect of genistein on a tumor promoter-induced c-fos and c-jun expression in mouse skin. *Oncol Reports* **3**: 125–128

Zava DT and Duwe G (1997) Estrogenic and antiproliferative properties of genistein and other flavanoids in human breast cancer cells *in vitro*. *Nutr Cancer* **27**: 31– 40

 *© 2001 Cancer Research Campaign*
